# Detection rates for prostate cancer using PI-RADS 2.1 upgrading rules in transition zone lesions align with risk assessment categories: a systematic review and meta-analysis

**DOI:** 10.1007/s00330-025-11618-w

**Published:** 2025-04-27

**Authors:** Georgios Agrotis, Eduardo Pais Pooch, Kostas Marsitopoulos, Marianna Vlychou, Matthias Benndorf, Regina G. H. Beets-Tan, Ivo G. Schoots

**Affiliations:** 1 Department of Radiology, The Netherlands Cancer Institute, Amsterdam, The Netherlands; 2 GROW School for Oncology and Reproduction, Maastricht University, Maastricht, The Netherlands; 3 Department of Radiology, University Hospital of Larissa, Larissa, Greece; 4 Department of Diagnostic and Interventional Radiology, Medical Faculty OWL, University of Bielefeld, Klinikum Lippe, Detmold, Germany; 5 Department of Radiology & Nuclear Medicine, Erasmus University Medical Center Rotterdam, Rotterdam, The Netherlands

**Keywords:** Prostate neoplasm, Clinically significant prostate cancer (csPCa), Magnetic imaging resonance (MRI), Diffusion-weighted imaging, Transition zone

## Abstract

**Objective:**

To evaluate and compare cancer detection rates (CDRs) of transition zone (TZ) lesions upgraded from PI-RADSv2.1 score 2 to 3 (“2 + 1”) or from 3 to 4 (“3 + 1”) using DWI and assess their clinical impact.

**Materials and methods:**

A systematic literature search was performed in Embase, Medline, and Web of Science for studies assessing TZ lesions with DWI in PI-RADSv2.1, with histology-confirmed grade group ≥ 2 cancer (GG ≥ 2) as the primary outcome. Risk of bias was evaluated using QUADAS-2. Pooled sensitivity, specificity, CDRs, and odds ratios (ORs) were estimated at the lesion level using a bivariate binomial random-effects model.

**Results:**

Eight studies with 1535 TZ lesions were included. GG ≥ 2 CDRs for PI-RADS scores of 1, 2, 2 + 1, 3, 3 + 1, 4, and 5 were 2% (95%CI: 0%–12%), 6% (4%–10%), 13% (6%–23%), 19% (15%–25%), 37% (24%–52%), 49% (32%–67%), and 73% (66%–79%), respectively. Scores of 2 + 1 had higher GG ≥ 2 CDRs than 2 (OR 3.37 (1.53–7.44), *p* = 0.003) but were similar to 3 (OR 0.80 (0.44–1.45), *p* = 0.46). Scores of 3 + 1 had higher GG ≥ 2 CDRs than 3 (OR 2.67 (1.27–5.59), *p* = 0.009) but were similar to 4 (OR 0.68 (0.33–1.44), *p* = 0.32). False-positive rates remained substantial (≥ 2 + 1: 69% (55%–80%); ≥ 3: 54% (46%–62%)).

**Conclusion:**

The risk of having significant prostate cancer in “2 + 1” and “3 + 1” TZ lesions, with an upgrading based on DWI images, is appropriately categorized within the PI-RADS v2.1 scoring system, as shown by this meta-analysis.

**Key Points:**

***Question*** *PI-RADS v2.1 incorporates rules allowing scores of some transition zone (TZ) lesions to be increased. Literature on the clinical impact of these rules is scarce.*

***Findings*** *For TZ lesions upgraded with DWI:* “*2* *+* *1*” *lesions show a cancer detection rate (CDR) of 13%, and* “*3* *+* *1*” *lesions show a CDR of 37%.*

***Clinical relevance**** Upgraded TZ lesions may impact individualized biopsy-decisions, especially as* “*3* *+* *1*” *lesions harbor significant disease in 2-out-of-5 patients. Still, the high rate of grade group* *=* *1 and benign findings in these sub-categories emphasizes the need for strategies to minimize overdiagnosis.*

**Graphical Abstract:**

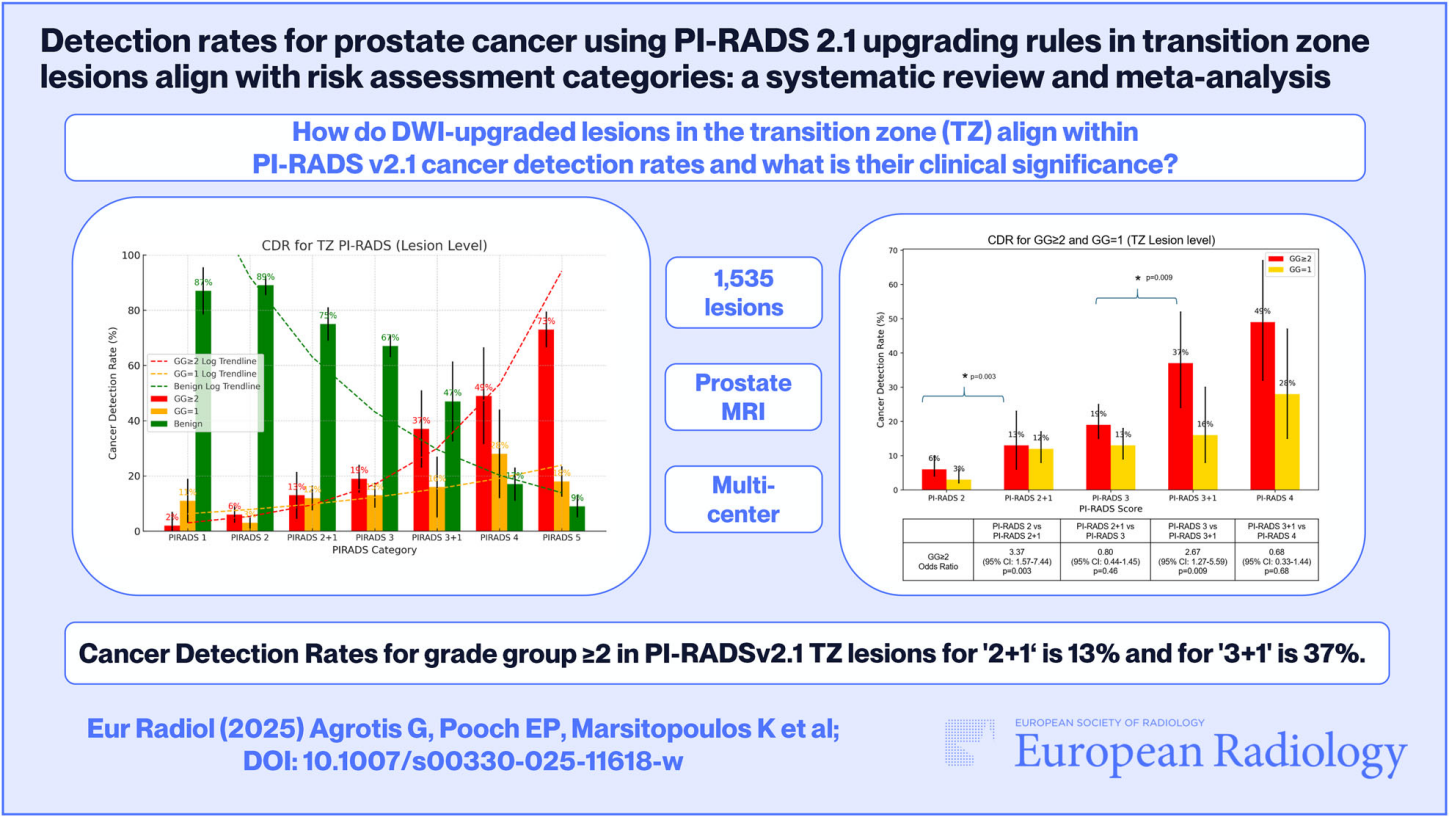

## Introduction

Magnetic resonance imaging (MRI) has become a widely utilized tool for the detection and risk stratification of prostate cancer (PCa). The Prostate Imaging Reporting and Data System version 2.1 (PI-RADS v2.1) has been established as a risk stratification tool for assessing suspicious findings on prostate MRI, particularly in estimating the likelihood of clinically significant prostate cancer (csPCa-GG ≥ 2). It places specific emphasis on diffusion-weighted imaging (DWI) for evaluating peripheral zone (PZ) lesions and T2-weighted imaging (T2WI) for transition zone (TZ) lesions [1].

**Fig. 1:**
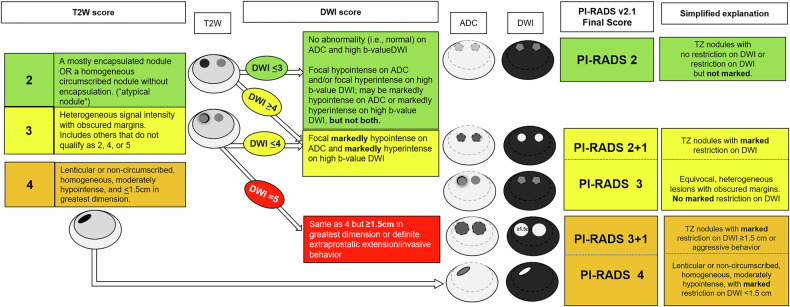
Schematic diagram shows the PI-RADS v2.1 upgrade rules for transition zone lesions and PI-RADS categories

To improve inter-reader agreement, PI-RADS v2.1 introduced important changes, particularly in the assessment of lesions in the TZ. In this version, the concept of “atypical nodules,” defined as a mostly encapsulated nodule or a homogeneous circumscribed nodule without encapsulation, was introduced. These “atypical nodules” can be upgraded from an overall score of 2 to 3 if there is associated marked hypointensity on ADC and marked hyperintensity on high *b*-value DWI. Here, “markedly” refers to a more pronounced signal change than any other focus in the same zone [1]. In contrast, the “upgrading rule” from score 3 to 4 was already present in PI-RADS v2. This rule considers not only the presence of marked restricted diffusion but also additional criteria such as a lesion diameter of ≥ 15 mm or evidence of extraprostatic extension (DWI score 5). These upgrading rules were designed to improve the accuracy and consistency of TZ lesion characterization, helping readers better understand and apply PI-RADS in clinical practice (Fig. 1).

These modifications may impact biopsy consideration in upgraded TZ lesions to category 3 or higher, emphasizing the importance of aligning diagnostic strategies to reduce false positives and minimize unnecessary biopsies.

This systematic review aims to evaluate the clinical implications of adhering to PI-RADS v2.1 upgrading rules, focusing on the benefit of DWI-based upgrades in MRI-guided diagnostic pathways for TZ lesions.

## Materials and methods

This systematic review was reported according to the Preferred Reporting Items for Systematic Review and Meta-Analysis (PRISMA) Studies checklists [2]. The review protocol is registered on the PROSPERO International Prospective Register of Systematic Reviews (registration no. CRD42024590422).

### Literature search

A systematic literature search was conducted in MEDLINE, EMBASE, and Web of Science databases to identify relevant articles. The “PICO” question to be addressed by this review was (P) Patients with suspected/proven PCa in the TZ zone; (I) Intervention—PI-RADS V2.1 scoring with DWI upgrades on TZ zone (“2 + 1” and “3 + 1”); (C) Comparison—PI-RADS scores without DWI upgrading in TZ nodules; (O) Outcome/Target Condition—diagnosis of high- or low-risk tumor based on histopathologic findings from either prostate biopsy or radical prostatectomy.

The complete search terms are provided in supplementary Text 1. The literature search was limited to English language publications and studies of human subjects. To retrieve additional publications, the reference lists of included articles and relevant reviews on the topic were assessed for potential inclusions. Finally, all citations were imported into a bibliographic database (EndNote X, Thomson Reuters).

### Study selection

After study retrieval, the title and abstract screens led to full article assessment when they met the following criteria: original research (no review articles, or case reports) and comprehensive TZ lesions assessment with DWI upgrades for the diagnosis of PCa, without previous treatments. For the meta-analysis, studies had to report on histologically proven PCa (reference, either from biopsy or radical prostatectomy), PI-RADS score v2.1, and provide sufficient data to calculate cancer detection rates for each score. *Study authors were contacted if critical data for quantitative analysis (meta-analysis) were missing.”*

### Data extraction

Two investigators (A.G., K.M.) independently extracted the required information for the calculation tables, reporting total number of patients, lesions, number of GG ≥ 1, GG ≥ 2, GG = 1 and benign lesions for each PI-RADS category, with special interest in the upgraded category 3 (category 2 + 1) and the upgraded category 4 (category 3 + 1). All analyses were conducted at the lesion level due to the unavailability of patient-level data from the studies.

In addition, patient and method characteristics were recorded for each study: author, year of publication, country, study design, reference standard, number of patients, history of prior prostate biopsy, scanner field strength, number of readers, and MRI manufacturer.

### Methodological quality assessment—publication bias

The methodological quality, potential introduction of bias, and applicability of the included studies in the meta-analysis were evaluated using the revised tool for the Quality Assessment of Diagnostic Accuracy Studies (QUADAS-2) [3]. Signaling questions specific to assessing the risk of bias are summarized in Supplemental Table 1. The knowledge of pathology findings (unblinded interpreting radiologist) obtained from biopsy or radical prostatectomy was considered a potential source of bias. A temporal separation longer than 6 months between MRI and biopsy/prostatectomy was deemed inappropriate. Biopsy alone was considered less likely to grade PCa accurately, and radical prostatectomy was regarded as the reference standard. Given the high heterogeneity among diagnostic accuracy studies, we performed the Deeks publication bias test [4], which was assessed by using the Deeks funnel plot asymmetry test.

### Statistical analysis

Random intercept logistic regression models for meta-analysis of single proportions were used to derive summary estimates for CDRs, as well as a random-effects model was used to calculate the pooled odds ratio (OR) across studies.

Summary estimates for sensitivity and specificity for all investigated scenarios were derived with bivariate binomial models [5]. SROC curves with 95% CIs were derived for the resulting point estimate of sensitivity and specificity.

Using the Deek test for diagnostic odds ratios, funnel plots were derived to analyze possible publication bias. *Q* statistic of the chi square value test and the inconsistency index (*I*^2^) were used to estimate the heterogeneity among included studies. Meta-regression analysis explored factors that contributed to the heterogeneity. Meta-regression was performed of pairwise combinations of selected study characteristics.

All analyses were performed using R (version 4.3.0; The R Foundation) with meta [6] and mada packages and RevMan 5.4.

## Results

### Study selection

**Fig. 2:**
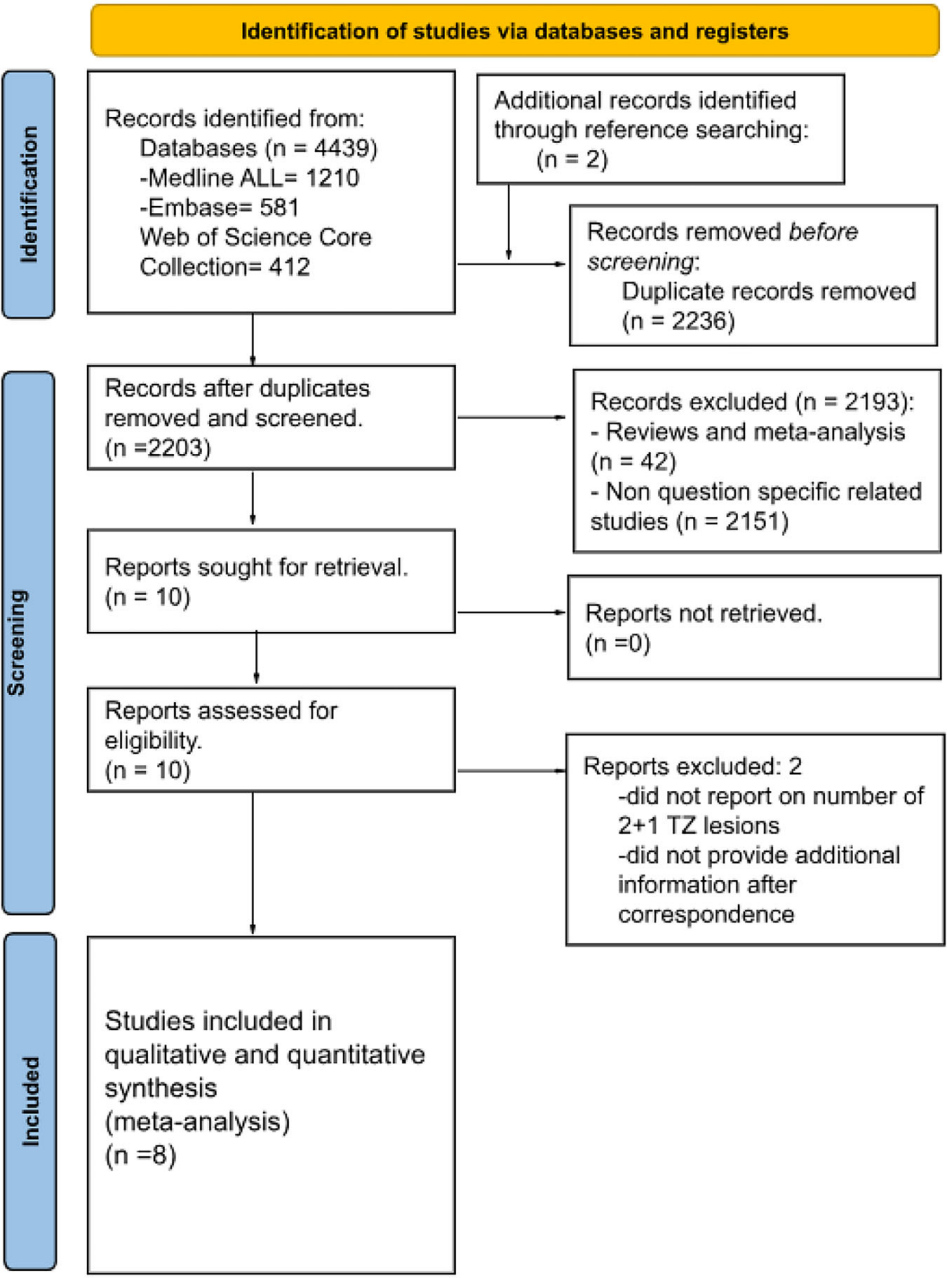
Flow diagram illustrating the systematic process of study selection, screening, eligibility assessment, and final inclusion of studies on the meta-analysis (PRISMA)

Following the PRISMA flow diagram (Fig. 2), 8 studies were included [7–14] with their demographic study (Suppl. Table 2), Clinical (Suppl. Table 3), and technical characteristics (Suppl. Table 4). Studies were published between 2020 and 2024; two of these adopted a prospective design, while six utilized a retrospective data collection.

### Overview of included studies

A total of 8 studies included 1535 TZ lesions, which included information on DWI upgraded lesions. Six studies used biopsy results, whereas two studies used radical prostatectomy results as the reference standard (Suppl. Table 3). The included studies had differences regarding MRI vendors, as well as readers’ experience (Suppl. Table 4). Key findings of the included studies are summarized in Table 1.

**Table 1:** Key findings—summary of PI-RADS studies on DWI upgraded lesions

Study	Key findings	Supporting data
Asai et al [10], 2024	Upgraded atypical nodules (2 + 1) had higher detection rates of PC and csPCa compared to non-upgraded; no significant difference from conventional category 3 lesions.	Detection rates: Upgraded nodules detected csPCa at a rate of 50.8%, compared to 16.2% in non-upgraded nodules. *p* < 0.05 for detection rate comparison.
Byun et al [12], 2020	PI-RADS v2.1 showed higher sensitivity and specificity (94.5% and 60.9%) than v2 (91.8% and 56.3%) for category ≥ 3 lesions in detecting csPCa, although not significantly. Of eight upgraded lesions from category 2 to 3 (2 + 1) with an incorporated DWI, 50% (4/8) were csPCa, significantly higher than category 2 lesions (4.4%; *p* = 0.003). No csPCa was detected among the 22.8% (46/201) downgraded lesions.	*p* = 0.003 for csPCa detection in upgraded lesions; Sensitivity: 94.5% (PI-RADSv2.1) vs. 91.8% (PI-RADSv2), Specificity: 60.9% (v2.1) vs. 56.3% (v2).
Rudolph et al [13], 2020	No significant difference between PI-RADS v2 and v2.1 in overall diagnostic performance for TZ lesions; DWI upgrade rule applied to a few TZ lesions; improved downgrading of benign TZ nodules (BPH).	AUC for TZ lesions: PI-RADS v2: 0.72, PI-RADS v2.1: 0.69; *p* > 0.05 for diagnostic performance; 0% csPCa in downgraded BPH nodules.
Lim et al [9], 2021	PI-RADS v2.1 DWI-upgraded TZ atypical nodule cancer rate is low, with 7.5% (3/40) showing clinically significant cancer. There was no difference in cancer detection rates between PI-RADS v2.1 category 3 upgraded TZ atypical nodules and conventional T2-weighted score 3 TZ nodules. PCa was not diagnosed in any atypical nodule that was not upgraded on DWI.	*p* = 0.09 for csPCa detection rates between DWI-upgraded nodules and conventional T2-weighted score 3 nodules. PCa detection rate: 7.5% for csPCa in DWI-upgraded nodules vs. 20% in conventional T2-weighted nodules.
Engel et al [14], 2022	Detection from consensus reading of PIRADS 2 + 1 was 1/7, from PI-RADS 3 + 0 was 0/18, for PI-RADS 3 + 1 was 2/5 and PI-RADS 4 + 0 was 4/12 for ISUP > 1.	Biopsy confirmed csPCa in 24/98 cases. Area under the curve (AUC) was 0.89/0.90 for reader 1, 0.92/0.91 for reader 2, and 0.92/0.91 for the consensus reading.
Yilmaz et al [7], 2023	Upgraded PI-RADS 3 TZ lesions were less likely to harbor csPCa compared with their non-upgraded counterparts.	csPCa detection rate: 4% in upgraded lesions (1 of 26) vs. 20% in non-upgraded lesions (20 of 99); *p* = 0.02.
Costa et al [8], 2021	No PCa, GG1 PCa, or csPCa was found in 84% (*n* = 41), 10% (*n* = 5), and 6% (*n* = 3) of 49 patients with 2 + 1 lesions who underwent targeted biopsy, nor in 74% (*n* = 45), 15% (*n* = 9), and 11% (*n* = 7) of 61 patients with 3 + 0 lesions. *p* = 0.31 for csPCa detection rate difference between 2 + 1 and 3 + 0 lesions.	csPCa detection rate: 6% in 2 + 1 lesions vs. 11% in 3 + 0 lesions (*p* = 0.31)
de Oliveira Correia et al [11], 2024	Frequencies of csPCa were not significantly different between dominant and upgraded PI-RADS 3 TZ lesions (20% vs. 19%), PI-RADS 4 TZ lesions (33% vs. 26%). Disregarding upgrading rules yielded 5.5 and 1.9 biopsies avoided per missed csPCa for MRI-focused and risk-based pathways.	This study supports the application of PI-RADS upgrading rules for optimizing patient selection for prostate biopsy, particularly in risk-based pathways.

### Quality assessment

QUADAS-2 assessment of patient selection showed an overall low risk of bias in all studies (8/8, 100%) (Suppl. Fig. 1). For the reference standard, all studies (8/8, 100%) showed a low risk of bias, as these studies used histology confirmation to validate their results (both targeted biopsy and radical prostatectomy were considered appropriate as the reference standard). The index test showed an overall low risk of bias in most of the studies (8/8, 100%), as the interpreting radiologists were blinded to the pathological records in those studies. More than half of the studies (5/8, 63.5%) demonstrated unclear flow and timing, with no report of a time gap of no more than 6 months between the biopsy/radical prostatectomy and the MRI. None of the included studies showed concerns regarding their applicability.

### Publication bias

Included studies were distributed asymmetrically in the scatterplot of diagnostic odds ratio against 1/(effective sample size)^1/2^ (Supp. Fig. 2). The result of the Deeks funnel plot asymmetry test (*p* = 0.08) showed no publication bias.

### Cancer detection rates for TZ lesions categorized into PI-RADS assessment scores

**Fig. 3:**
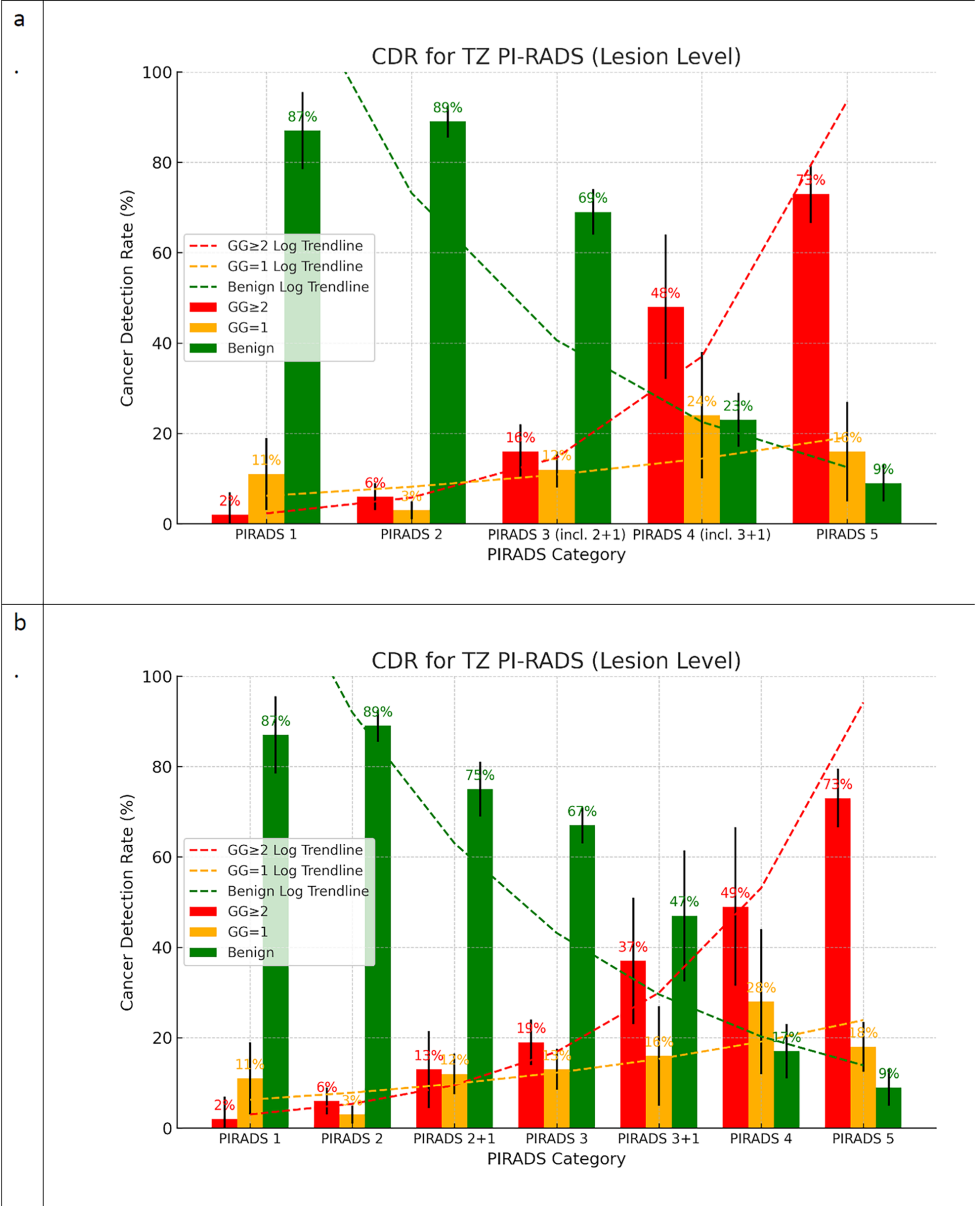
**a**, **b** Cancer detection rates (CDR) for GG = 1, GG ≥ 2, benign TZ lesions with their corresponding 95% confidence intervals and trend lines for (**a**) PIRADS 1-5, (**b**) PI-RADS 1-5 with categories “2 + 1” and “3 + 1” separated

603 lesions (39%) were positive for PCa, and 386 (25%) were positive for GG ≥ 2. CDRs at the lesion level for GG ≥ 2 were as follows: PI-RADS 1, 2%; PI-RADS 2, 6%; PI-RADS 2 + 1, 13%; PI-RADS 3, 19%; PI-RADS 3 + 1, 37%; PI-RADS 4, 49%; and PI-RADS 5, 73%. The CDR analysis for GG ≥ 1 as the outcome variable at the lesion level showed 13%, 10%, 25%, 33%, 53%, 83%, and 91% for PI-RADS 1–5, respectively. The CDR analysis for GG = 1, as the outcome variable at the lesion level showed 11%, 3%, 12%, 13%, 16%, 28%, and 18% for PI-RADS 1–5, respectively (Table 2 and Fig. 3; Sup. Fig. 3).

**Table 2:** Cancer detection rates of TZ PI-RADS categories at the lesion level

PI-RADS category and outcome	No. of studies analyzed	No. of lesions	No. of positive lesions	CDR summary estimate (%)	*I*² statistic, *p*-value
GG ≥ 2					
PI-RADS 1	4	64	1	2 (0, 12)	0, 1.00
PI-RADS 2	7	281	18	6 (4, 10)	0, 1.00
PI-RADS 2 + 1	8	208	28	13 (6, 23)	0.56, 0.03
PI-RADS 3	8	599	122	19 (15, 25)	0.22, < 0.01
PI-RADS 3 + 1	4	43	16	37 (24, 52)	0, 0,52
PI-RADS 4	5	163	71	49 (32, 67)	0.47, < 0.01
PI-RADS 5	4	177	130	73 (66, 79)	0, 0.57
GG ≥ 1					
PI-RADS 1	4	64	8	13 (6, 23)	0, 0.83
PI-RADS 2	7	281	27	10 (7, 14)	0, 0.85
PI-RADS 2 + 1	8	208	53	25 (20, 32)	0.10, 0.35
PI-RADS 3	8	599	196	33 (29, 37)	0.45, 0.08
PI-RADS 3 + 1	4	43	23	53 (39, 68)	0, 0.71
PI-RADS 4	5	163	135	83 (76, 88)	0.33, 0.2
PI-RADS 5	4	177	161	91 (86, 94)	0, 0.77

Data in parentheses are 95% CIs

*CDR* cancer detection rate, *GG* grade group, *PI-RADS* Prostate Imaging Reporting and Data System

### Odds ratio for Subcategories PI-RADS 2 + 1 and 3 + 1

**Fig. 4:**
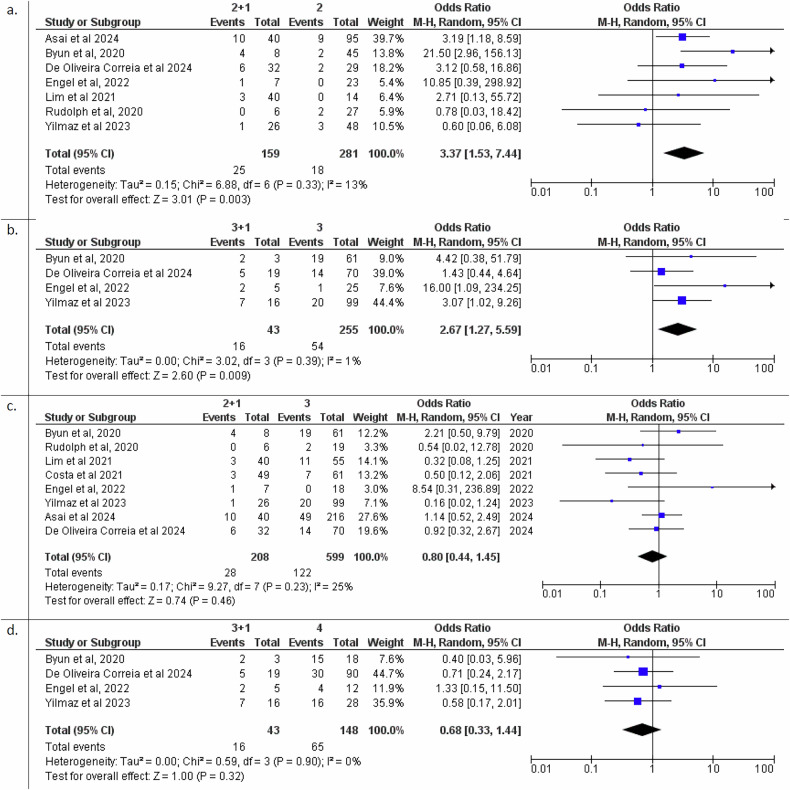
**a**–**d** Forest Plots for Odds Ratios of (**a**) PIRADS 2 + 1 vs. 2, (**b**) 3 + 1 vs. 3, (**c**) 2 + 1 vs. 3, (**d**) 3 + 1 vs. 4 in predicting GG ≥ 2

The comparison between PI-RADS 2 and PI-RADS 2 + 1 shows a significant increase in the odds of GG ≥ 2 cancer for PI-RADS 2 + 1 (OR: 3.37 (95% CI: 1.57–7.44)), indicating that applying the DWI upgrade rule is associated with a higher likelihood of significant findings. In contrast, the comparison between PI-RADS 2 + 1 and PI-RADS 3 does not reveal a significant difference (OR: 0.80 (95% CI: 0.44–1.45)), suggesting similar ORs between these two categories. However, the comparison between PI-RADS 3 and PI-RADS 3 + 1 indicates that PI-RADS 3 + 1 category has significantly higher odds ((OR: 2.67, 95% CI: 1.27–5.59)), reinforcing the value of the DWI-upgrading rule in distinguishing more GG ≥ 2 cancer. Categories PI-RADS 3 + 1 and 4 were not statistically different (OR: 0.68 (95% CI: 0.33–1.44)), suggesting that both categories may reflect a similar level of risk (Fig. 4a–d).

### Diagnostic accuracy

**Fig. 5:**
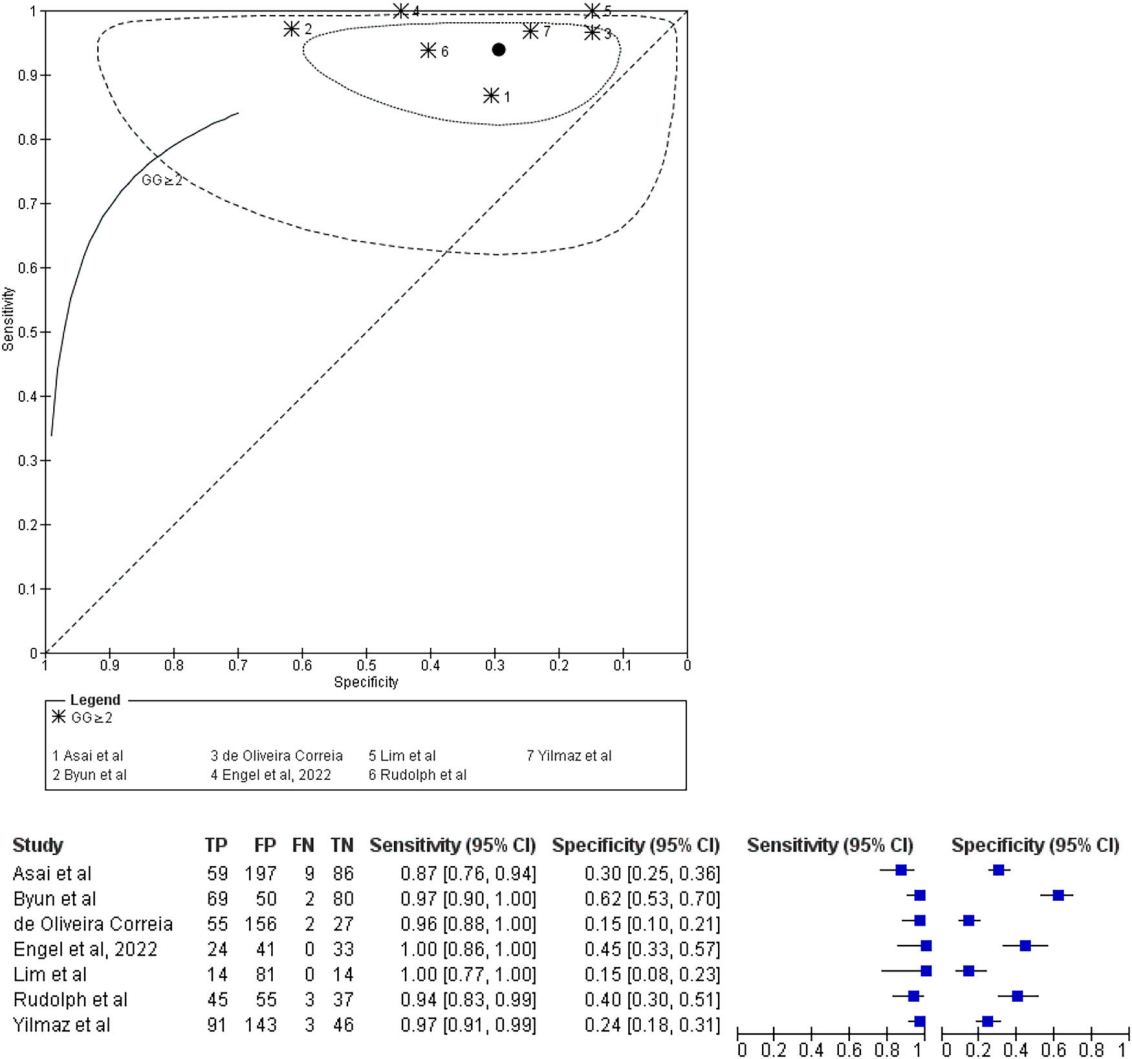
Diagnostic accuracy with Prostate Imaging Reporting and Data System (PI-RADS) greater than or equal to 2 + 1 considered positive and GG ≥ 2 as the outcome. Summary receiver operating characteristic (SROC) curve shows diagnostic accuracy at the lesion level (area under the SROC curve, 0.86(95% CI: 0.82–0.90) and corresponding forest plots show sensitivity and specificity, with 94% (95% CI: 90%–97%) and 31% (95% CI: 20%–45%), respectively

At the lesion level, thresholding at PI-RADS ≥ 2 + 1 yielded a sensitivity of 94% (95% CI: 90%–97%), specificity of 31% (95% CI: 20%–45%), and an area under the SROC curve of 0.86 (95% CI: 0.82–0.90) for detecting GG ≥ 2 disease. For GG ≥ 1 disease, sensitivity and specificity were 93% (95% CI: 89%–96%) and 37% (95% CI: 26%–50%), with an AUC of 0.84 (95% CI: 0.80–0.89) (Table 3 and Fig. 5). When applying a PI-RADS ≥ 3 threshold (excluding 2 + 1), the sensitivity, specificity, and AUC for csPCa detection were 88% (95% CI: 79%–93%), 46% (95% CI: 38%–54%), and 0.69 (95% CI: 0.63–0.75), respectively. For detecting any PCa at this threshold, these values were 85% (95% CI: 76%–91%), 47% (95% CI: 36%–59%), and 0.73 (95% CI: 0.67–0.79). False positive rates were substantial in both threshold outcomes for GG ≥ 2 detection (≥ 2 + 1: 69% (95% CI: 55%–80%) and ≥ 3: 54% (95% CI: 46%–62%)) (Table 3).

**Table 3:** Meta-analysis of diagnostic accuracy on a TZ lesion level stratified according to PI-RADS threshold, and outcome

Scenario investigated	No. of studies analyzed	No. of lesions	No. of positive lesions	Sensitivity (95% CI)	Specificity (95% CI)	AUC (95% CI)	False-positive rate (95% CI)
PI-RADS ≥ 2 + 1 considered positive for GG ≥ 2	7	1425	376	0.94 (0.90–0.97)	0.31 (0.20–0.45)	0.86 (0.82–0.90)	0.69 (0.55–0.80)
PI-RADS ≥ 2 + 1 considered positive for GG ≥ 1	7	1425	579	0.93 (0.89–0.96)	0.37 (0.26–0.50)	0.84 (0.80–0.89)	0.63 (0.50–0.74)
PI-RADS ≥ 3 (but not 2 + 1) considered positive for GG ≥ 2	8	1535	386	0.88 (0.79–0.93)	0.46 (0.38–0.54)	0.69 (0.63–0.75)	0.54 (0.46–0.62)
PI-RADS ≥ 3 (but not 2 + 1) considered positive for GG ≥ 1	8	1535	603	0.85 (0.76–0.91)	0.47 (0.36–0.59)	0.73 (0.67–0.79)	0.53 (0.41–0.65)
PI-RADS ≥ 3 + 1 considered positive for GG ≥ 2	4	387	246	*			

Data in parentheses are 95% CIs

*GG* grade group, *PI-RADS* Prostate Imaging Reporting and Data System, *SROC* summary receiver operating characteristic

* Due to the small number of studies, DTA analysis could not be performed

### Meta-regression analysis

Due to data availability, meta-regression analysis was limited to age (cutoff: 65 years) and PSA density (threshold: 0.15), indicating no significant impact on heterogeneity across studies. For age, the coefficient was −0.224 (95% CI: −2.975 to 2.527, *p* = 0.812), and for PSA density, −0.558 (95% CI: −4.200 to 3.083, *p* = 0.577), suggesting neither variable substantially influenced pooled effect estimates.

## Discussion

This systematic review and meta-analysis aimed to assess the diagnostic performance and clinical implications of upgrading TZ lesions in prostate MRI using diffusion-weighted imaging in PI-RADS v2.1 scoring. Our analysis included eight studies, incorporating a total of 1535 TZ lesions. The results provide important insights into the cancer detection rates for TZ lesions upgraded from PI-RADS 2 to 2 + 1 and from PI-RADS 3 to 3 + 1 using DWI, as well as the clinical significance of these upgrades.

One of the most notable findings is that the CDR for lesions GG ≥ 2, increased from 6% for PI-RADS 2 lesions to 13% for PI-RADS 2 + 1 lesions (OR 3.37, *p* = 0.003), demonstrating the value of DWI upgrading for distinguishing clinically relevant cases. Similarly, the CDR for PI-RADS 3 + 1 lesions (37%) was significantly higher than for PI-RADS 3 lesions (19%) (OR 2.67, *p* = 0.009), further validating the effectiveness of the DWI upgrading rule in improving cancer detection.

Upgrading atypical nodules from category “2” to “2 + 1” in prostate imaging has shown variable outcomes across the included studies, highlighting both potential benefits and limitations. Specifically, Asai et al demonstrated that DWI upgrading significantly increased the detection rate of csPCa to 50.8%, compared to 16.2% in non-upgraded nodules (*p* < 0.05), though detection rates were similar to those of category “3” lesions. Similarly, Byun et al reported that 50% of upgraded “2 + 1” lesions were csPCa, markedly higher than the 4.4% in category “2” nodules (*p* = 0.003). In contrast, other studies found no significant difference between upgraded “2 + 1” lesions and conventional category “3” nodules. For instance, Lim et al noted comparable detection rates between these groups (*p* = 0.09), while de Oliveira Correia found no significant difference in csPCa frequency between dominant and upgraded PI-RADS “3” and “4” lesions. Additionally, Yilmaz et al reported that upgraded “2 + 1” nodules were less likely to harbor csPCa compared to non-upgraded “3” lesions (*p* = 0.02), and Costa et al observed no significant improvement in csPCa detection with upgrading (*p* = 0.31). These findings collectively underscore the variability in the diagnostic utility of DWI upgrading, highlighting the need for clearer categorization and more precise clinical management of upgraded nodules in prostate imaging.

In 2017, Rosenkrantz et al showed [15] that upgrading TZ lesions from PI-RADS category 3 to 4 based on a DWI score of 5 effectively detected GS ≥ 7 in 50.0%–66.7% of cases. They also proposed adjustments to upgrade TZ lesions based on a DWI score of 4 where they estimated tumor detection rates for GS ≥ 7, ranging from 30%–60%, highlighting the potential value of using lower DWI thresholds to improve the sensitivity of the previous version PI-RADSv2 in identifying GG ≥ 2 in the TZ. In 2018, Thai et al [16] found significantly higher cancer rates for upgraded T2-weighted MRI score “3 + 1” nodules using DWI compared with conventional PI-RADS v2 category 3 nodules (30.8% vs. 11.1%, respectively).

In a recent meta-analysis by Oerther et al [17], dedicated to benchmark PI-RADS assessment categories, GG ≥ 2 CDRs for PI-RADS 2 + 1 and 3 subcategory TZ lesions were 12% (95% CI: 7–19%) and 19% (95% CI: 12–29%), based on 146 lesions and 155 lesions, respectively, whereas in our meta-analysis we incorporated 208 and 599 lesions, respectively, alongside 43 PI-RADS 3 + 1 lesions, a category not previously quantitatively analyzed in a meta-analysis. The current meta-analysis validates the previously hypothesized distinctions between the PI-RADS categories and the 2 + 1 and 3 + 1 categories, as demonstrated in Fig. 3a, b, where these categories serve as an intermediate step between their adjacent categories.

The low specificity values (31%–47%), observed within the different threshold categories, highlight a critical challenge in the current diagnostic approaches. While sensitivity remains a key priority to ensure csPCa are not missed, the trade-off with specificity can lead to overdiagnosis and unnecessary biopsies, which may increase patients’ stress and healthcare costs. This is particularly evident in the analysis of upgraded lesions (e.g., “2 + 1”) and lower-threshold categorizations, where the emphasis on capturing more csPCa inadvertently increases the amount of indolent disease being found, with a “harm to benefit ratio” of 0.9 (supplementary Fig. 3). These findings suggest the need to refine imaging criteria and incorporate additional risk factors, to enhance specificity without compromising sensitivity. Future studies could also explore integrating quantitative imaging biomarkers or machine learning algorithms to improve the balance between these diagnostic metrics. The clinical impact of these findings is significant, particularly when considering individualized biopsy decisions. TZ lesions scored as PI-RADS 3 + 1 exhibit a cancer detection rate similar to that of PI-RADS 4 lesions, with 2-in-5 harboring clinically significant disease. This suggests that TZ lesions upgraded to 3 + 1 warrant close clinical attention and should be considered for biopsy in the same manner as PI-RADS 4 lesions. On the other hand, despite the increased GG ≥ 2 CDR in PI-RADS 2 + 1 lesions than PI-RADS 2, detecting nearly as many GG1 as GG ≥ 2 cancers raises concern, and efforts should be made to avoid overtreatment.

Based on our findings, omitting biopsies in the 2 + 1 subcategory would save approximately 8 unnecessary biopsies for each missed GG ≥ 2 cancer, while omitting biopsies in the 3 + 1 subcategory would save only approximately three biopsies per missed GG ≥ 2 cancer. Similarly, de Oliveira Correia et al [13] showed that using MRI-focused pathways and disregarding upgrading rules yielded 5.5 biopsies avoided per missed GG ≥ 2 cancer. In other words, “2 + 1” category impacts a higher biopsy avoidance, whereas “3 + 1” category impacts GG ≥ 2 cancer detection. These findings underscore the need to carefully balance the benefit of reducing unnecessary biopsies with the risk of missing significant cancers in MRI-focused diagnostic pathways, thereby emphasizing the need for personalized biopsy strategies and incorporating quantitative biomarkers to reduce false positives [18].

There are several limitations to our analysis that must be acknowledged. The inclusion of only eight studies, coupled with varying MRI machines and radiologist experience across studies, introduces moderate heterogeneity (*I*² < 50%). Efforts to reach more authors for supplementary data were undertaken, but still, the data were limited. While our analysis is lesion-based, which aligns with data availability of the included studies, it has limitations for clinical decision-making. For example, a single “2 + 1” nodule in the TZ may have little impact if a category 4 or 5 lesion is also present, as the patient would likely proceed to biopsy based on the higher-risk lesion. Additionally, our lesion-based analysis does not account for how higher PI-RADS lesions might influence the cancer risk of lower-grade lesions within the same patient. Given the multifocal nature of PCa, the likelihood of clinically significant outcomes in a lower-grade lesion could increase when a higher-grade lesion is also present. We could not analyze GG ≥ 3 lesions due to data unavailability, limiting our assessment of higher-grade PCas. This gap highlights the need for further studies to explore different threshold levels for biopsy decision-making, especially GG3 + . Future research comparing lesion and patient-level findings, as well as multifocal lesion,s could improve the clinical applicability of PI-RADS upgrades and biopsy decision making.

## Conclusions

The risk of having significant cancer in “2 + 1” and “3 + 1” lesions aligns within the PI-RADS scoring system and appears to enhance overall sensitivity. However, this comes at the cost of an increased rate of false positives. These findings have important implications for biopsy decision-making in patients with TZ lesions and underline the need for personalized biopsy decision-making to reduce overdiagnosis and overtreatment.

## Supplementary information

ELECTRONIC SUPPLEMENTARY MATERIAL

## Abbreviations

DWI: Diffusion-weighted imaging
GG ≥ 2: Higher than grade group 2
GG1: grade group 1
PCa: Prostate cancer
TZ: Transition zone
