# Supplementary material for: Detection rates for prostate cancer using PI-RADS 2.1 upgrading rules in transition zone lesions align with risk assessment categories: a systematic review and meta-analysis
Source: Eur Radiol. 2025 Apr 27;35(10):6454–65. doi: 10.1007/s00330-025-11618-w (PMC12417277; doi:10.1007/s00330-025-11618-w)
Supplement: Supplementary file 1 — ELECTRONIC SUPPLEMENTARY MATERIAL [file 330_2025_11618_MOESM1_ESM.pdf]

Detection rates for Prostate Cancer using PI-RADS 2.1 upgrading rules in transition zone lesions align with risk assessment categories: a systematic review and meta-analysis

ELECTRONIC SUPPLEMENTARY MATERIAL

Eur Radiol (2025) Agrotis G, Pooch EP, Marsitopoulos K, et al.

Supplementary Text 1.

Database searched	Platform	Years of coverage	Records	Records after duplicates removed
Medline ALL	Ovid	1946 - Present	1215	1210
Embase	Embase.com	1971 - Present	1722	581
Web of Science Core Collection*	Web of Knowledge	1975 - Present	1502	412
**Total**			**4439**	**2203**

*Science Citation Index Expanded (1975-present) ; Social Sciences Citation Index (1975-present) ; Arts & Humanities Citation Index (1975-present) ; Conference Proceedings Citation Index- Science (1990-present) ; Conference Proceedings Citation Index- Social Science & Humanities (1990-present) ; Emerging Sources Citation Index (2005-present)

No other database limits were used than those specified in the search strategies

medline 1215

(((prostat*-imag* ADJ3 report* ADJ3 data ADJ3 system*) OR pirads* OR pi-rads*).ab,ti,kw.) AND (Diffusion Magnetic Resonance Imaging / OR Multiparametric Magnetic Resonance Imaging / OR ((diffusion ADJ3 weighted ADJ3 imag*) OR ((multi-paramet* OR bi-paramet* OR multiparamet* OR biparamet*) ADJ3 (mri OR mr OR magnet*-resonan* OR resonan*-imag*)) OR mpmri OR dwi).ab,ti,kw.) AND 2019:2025.(sa_year). NOT (exp animals/ NOT humans/) AND english.la.

embase 1722

('prostate imaging reporting and data system'/de OR ((prostat*-imag* NEAR/3 report* NEAR/3 data NEAR/3 system*) OR pirads* OR pi-rads*):Ab,ti,kw) AND ('diffusion weighted imaging'/de OR 'multiparametric magnetic resonance imaging'/de OR 'biparametric magnetic resonance imaging'/de OR ((diffusion NEAR/3 weighted NEAR/3 imag*) OR ((multi-paramet* OR bi-paramet* OR multiparamet* OR biparamet*) NEAR/3 (mri OR mr OR magnet*-resonan* OR resonan*-imag*)) OR mpmri OR dwi):ab,ti,kw) AND [2019-2025]/py NOT [conference abstract]/lim NOT ([animals]/lim NOT [humans]/lim) AND [english]/lim

Web of science 1502

TS=((((prostat*-imag* NEAR/2 report* NEAR/2 data NEAR/2 system*) OR pirads* OR pi-rads*)) AND (((diffusion NEAR/2 weighted NEAR/2 imag*) OR ((multi-paramet* OR bi-paramet* OR multiparamet* OR biparamet*) NEAR/2 (mri OR mr OR magnet*-resonan* OR resonan*-imag*)) OR mpmri OR dwi))) AND DT=(article) AND LA=(english)

Supplemental Table 1. Criteria for assessing risk of bias used in this study.

**Patient selection**
1.1 Was a consecutive or random sample of patients enrolled?
1.2 Was a case-control design avoided?
1.3 Did the study avoid inappropriate exclusions?
**Index Test**
2.1 Were the index test results interpreted without knowledge of the results of the reference standard? *(Knowledge of pathology findings from biopsy/radical prostatectomy was considered as a source of bias)*
2.2 If a threshold was used, was it pre-specified?
**Reference Standard**
3.1 Is the reference standard likely to correctly classify the target condition? *(biopsy was considered likely to grade prostate cancer correctly for purposes of this metanalysis, therefore both biopsy and radical prostatectomy were considered appropriate)*
3.2 Were the reference standard results interpreted without knowledge of the results of the index test?
**Flow and Timing**
4.1 Was there an appropriate interval between index tests and reference standard? *(temporal separation longer than 6 months was considered inappropriate)*
4.2 Did all patients receive a reference standard?
4.3 Did all patients receive the same reference standard?
4.4 Were all patients included in the analysis?

**Suppl. TABLE 2. DEMOGRAPHIC AND STUDY CHARACTERISTICS**						
**Study**	**Year**	**Study Type**	**Country**	**Journal**	**Biopsy technique**	**Reference standard**
Rudolph et al(13)	2020	Retrospective	Germany	Scientific Reports	targeted MRI/TRUS fusion biopsy + systematic biopsy	targeted biopsy
Byun et al (12)	2020	Retrospective	South Korea	Journal of Magnetic Resonance Imaging	Not specified	radical prostatectomy
Costa et al (8)	2021	Prospective	USA	American Journal of Roentgenology	MRI-TRUS fusion biopsy, MRI-guided in-bore biopsy	radical prostatectomy
Lim et al (9)	2021	Retrospective	Canada	American Journal of Roentgenology	cognitive fusion TRUS-guided biopsy	targeted biopsy
Engel et al (14)	2022	Retrospective	Germany	In Vivo	Transperineal MRI-TRUS fusion biopsy	targeted biopsy
Yilmaz et al (7)	2023	Prospective	USA	Radiology	MRI/US-guided biopsy and systematic biopsy	targeted biopsy
de Oliveira Correia et al(11)	2024	Retrospective	USA	American Journal of Roentgenology	MRI/TRUS fusion	targeted biopsy
Asai et al(10)	2024	Retrospective	Japan	International Journal of Urology	MRI/ultrasound fusion targeted biopsy	targeted biopsy

**Suppl. TABLE 3. Clinical Characteristics of Studies**						
**Study**	**Patient Sample Size**	**Mean Age (Years)**	**Mean PSA (ng/mL)**	**Prostate volume (mL)**	**PSAD (ng/mL ^{2})**	**Biopsy naïve**
Rudolph et al(13)	333	66.8 ± 7.4	12.8 ± 11.7	62.8 ± 32.1	Not specified	Not specified
Byun et al (12)	142	67 ± 7.0	8.33 ± 7.81	32.83 ± 11.77	Not specified	Not specified
Costa et al(8)	103	65.3 ± 5.7	7.4 ± 3.6	66.4 ± 31.2	0.14 ± 0.1	No
Lim et al (9)	104	64.8 ± 8.4	10.6 ± 7.2	71.0 ± 46.1	0.177 ± 0.113	No
Engel et al (14)	85	66.2 ± 7.7	12.2 ± 8.8	76 ± 40	Not specified	Not specified
Yilmaz et al(7)	454	67 ± 4.0	6.7 ± 2.7	59 ± 20.5	0.11 ± 0.05	no
de Oliveira Correia et al (11)	716	64.9 ± 8.4	6.1 ± 2.35	51.3 ± 28.1	0.14 ± 0.055	No
Asai et al(10)	321	68 ± 10.2 [	8.6 ± 19.1	Not specified	0.19 ± 0.85	Not specified
Note: PSA = prostate-specific antigen, PSAD= prostate specific antigen density						

Suppl. TABLE 4. Imaging and Readers Characteristics						
**Study**	**Field Strength**	**Vendor**	**Endorectal Coil Used**	**Spasmolytic Agent Used**	**Number of Radiologists**	**Radiologist Experience**
Rudolph et al(13)	3 Tesla	Siemens	Not specified	Not specified	3	>5 years
Byun et al (12)	3 Tesla	Philips, Siemens	No	Yes	3	>3 years
Costa et al(8)	3 Tesla	Philips Healthcare	No	Not specified	11	5–20 years of experience
Lim et al (9)	3 Tesla	Philips Healthcare	Not specified	Not specified	3	2, 7, and 7 years of experience
Engel et al (14)	3 Tesla	Siemens	No	Yes	2	3 and 7 years of experience
Yilmaz et al(7)	3 Tesla	Philips Healthcare	Yes	Not specified	1	>15 years
de Oliveira Correia et al (11)	1.5T and 3T	Siemens, GE, Philips	No	Yes	More than 20	15 years for key radiologists
Asai et al(10)	Not specified	Not specified	Not specified	Not specified	Not specified	experienced

Supplemental Figure 1. Graphical display of QUADAS-2 results by proportion of low, high, or unclear bias or applicability.

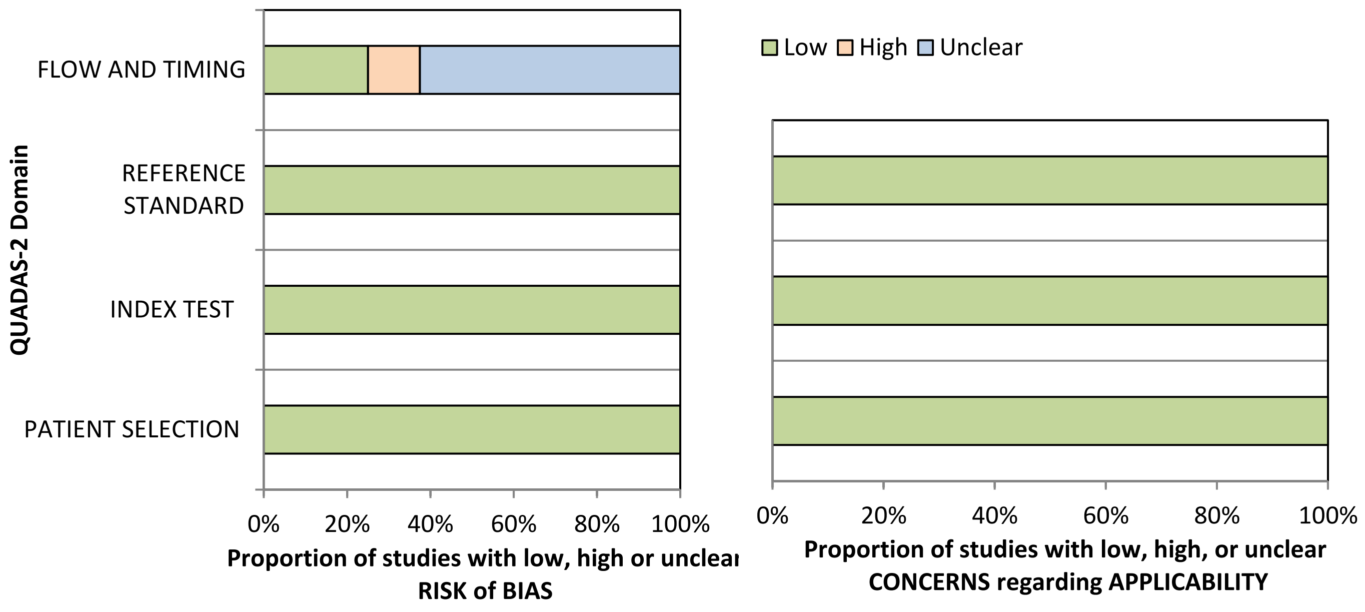

Supplemental Figure 2. Publication Test bias – Funnel Plot

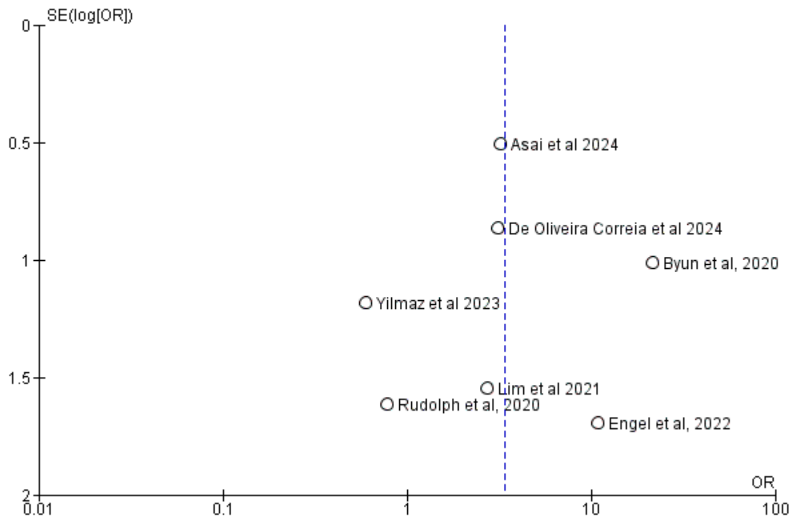

Supplemental Figure 3. Cancer Detection Rates (CDR) for GG=1 and GG≥2, with their corresponding 95 confidence intervals for PI-RADS 2-4 with categories '2+1' and '3+1' separated and Odds Ratios of PIRADS 2+1 vs 2, 3+1 vs 3, 2+1 vs 3, 3+1 vs 4 in predicting GG≥2. * symbol indicates statistical difference for GG≥2 detection between groups.

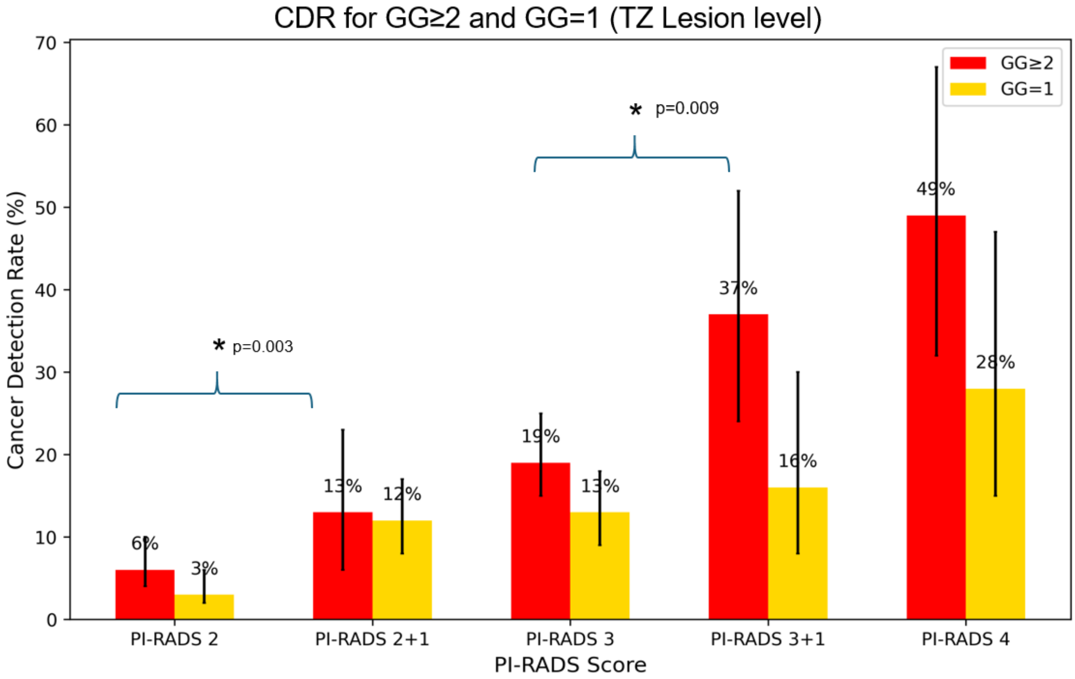

	PI-RADS 2 vs PI-RADS 2+1	PI-RADS 2+1 vs PI-RADS 3	PI-RADS 3 vs PI-RADS 3+1	PI-RADS 3+1 vs PI-RADS 4
GG≥2 Odds Ratio	3.37 (95% CI: 1.57-7.44) p=0.003	0.80 (95% CI: 0.44-1.45) p=0.46	2.67 (95% CI: 1.27-5.59) p=0.009	0.68 (95% CI: 0.33-1.44) p=0.68
